# A study protocol of a randomised controlled trial to measure the effects of an augmented prescribed exercise programme (APEP) for frail older medical patients in the acute setting

**DOI:** 10.1186/s12877-016-0252-z

**Published:** 2016-04-08

**Authors:** Ruth McCullagh, Eimear O’Connell, Sarah O’Meara, Ivan Perry, Anthony Fitzgerald, Kieran O’Connor, N. Frances Horgan, Suzanne Timmons

**Affiliations:** Centre for Gerontology & Rehabilitation, University College Cork, Cork, Ireland; Physiotherapy Department, Mercy University Hospital, Cork, Ireland; Clinical Research Facility, Mercy University Hospital, Cork, Ireland; Epidemiology & Public Health, University College Cork, Cork, Ireland; Department of Geriatric Medicine, Mercy University Hospital, Cork, Ireland; School of Physiotherapy, Royal College of Surgeons in Ireland, Dublin, Ireland

**Keywords:** Frail, Medical, Inpatients, Exercise, Physiotherapy, Length of stay

## Abstract

**Background:**

Older adults experience functional decline in hospital leading to increased healthcare burden and morbidity. The benefits of augmented exercise in hospital remain uncertain. The aim of this trial is to measure the short and longer-term effects of augmented exercise for older medical in-patients on their physical performance, quality of life and health care utilisation.

**Design & Methods:**

Two hundred and twenty older medical patients will be blindly randomly allocated to the intervention or sham groups. Both groups will receive usual care (including routine physiotherapy care) augmented by two daily exercise sessions. The sham group will receive stretching and relaxation exercises while the intervention group will receive tailored strengthening and balance exercises. Differences between groups will be measured at baseline, discharge, and three months. The primary outcome measure will be length of stay. The secondary outcome measures will be healthcare utilisation, activity (accelerometry), physical performance (Short Physical Performance Battery), falls history in hospital and quality of life (EQ-5D-5 L).

**Discussion:**

This simple intervention has the potential to transform the outcomes of the older patient in the acute setting.

**Trial registration:**

ClinicalTrials.gov Identifier: NCT02463864, registered 26.05.2015.

## Background

### Older medical patients can experience a prolonged acute hospital stay and functional decline

In Ireland in 2011, 11.6 % of the population was aged 65 years and over [1], and this is set to rise to 22 % by 2041 [2]. Extended periods of poor health are predicted with this longevity [2]. Older patients occupy most acute hospital beds and most frequently experience a prolonged length of stay (of greater than 30 days) [3], functional decline, high re-admission rates, falls, and institutionalisation [4]. Frailty is described as a geriatric syndrome with reduced capacity of the individual to resist stress and includes characteristics of slow mobility, low physical activity (PA) and energy levels [5]. Inactivity has been identified as a major determinant in the onset of frailty and exercise has been found to prevent or slow down this decline [6]. Therefore, maintenance of older adults’ functional independence while in hospital is of utmost importance.

### Physical activity levels and exercise intervention for medical patients in hospital

Recent evidence has shown that older medical patients walk an average of 1534 (±112) steps per day in hospital and that prolonged length of stay was inversely associated with daily step count, even when adjusted for age, gender and physical performance on admission [7]. Similarly, Fisher et al. [8] found that older adults, who increase their walking activity by 600 steps on the second day of observation, were discharged home two days earlier. These findings suggest that low physical activity in hospital may directly influence length of stay and supports the theory that patients should exercise and remain active in the acute setting. Exercise programmes in hospital have been delivered independently or as a component of a multidisciplinary intervention and have been shown to improve physical performance, quality of life, reduce falls incidence and reduce healthcare utilisation [9–13].

### The evidence of effectiveness of augmented exercise in hospital

To date, small benefits from augmented exercise on function and healthcare utilisation have been found. A systematic review found limited benefits from exercise as part of a multidisciplinary service on function, length of stay and discharge destination for acutely hospitalised older medical in-patients [14]. Three trials investigated the benefits of additional exercise alone [15–17]; none of which showed a significant improvement on length of stay. The authors suggested that the findings might have been weakened by using inappropriate outcome measures, recruitment of patients who had good baseline physical performance levels, and poor adherence to the exercise intervention that was being prescribed.

### Rationale for the trial and protocol

To date, additional exercise has not been found to shorten frail older patients’ hospital stay but the issues reported by previous authors may have weakened the results. To address these issues, the proposed protocol differs from previous studies in key parameters. A qualified physiotherapist will deliver and support the exercise sessions. Only patients who are less able to maintain physical activity will be recruited; those who need a walking aid and/or physical assistance on admission. Those who are unable to walk with assistance will be excluded from the trial. The Short Physical Performance Battery (SPPB) and walking speed will be used to measure physical performance, as these were previously found to be sensitive and appropriate for the study group [13]. The control arm will include sham exercises, to control for the considerable increase in patient-physiotherapist contact time. Finally, independent physical activity (usually walking) will be promoted outside the exercise sessions, in the intervention group.

Therefore, the aim of this study is to measure the effects of an augmented prescribed exercise programme for frail older medical inpatients on their physical performance, quality of life and healthcare utilisation.

## Methods

### Design and study size

**Fig. 1:**
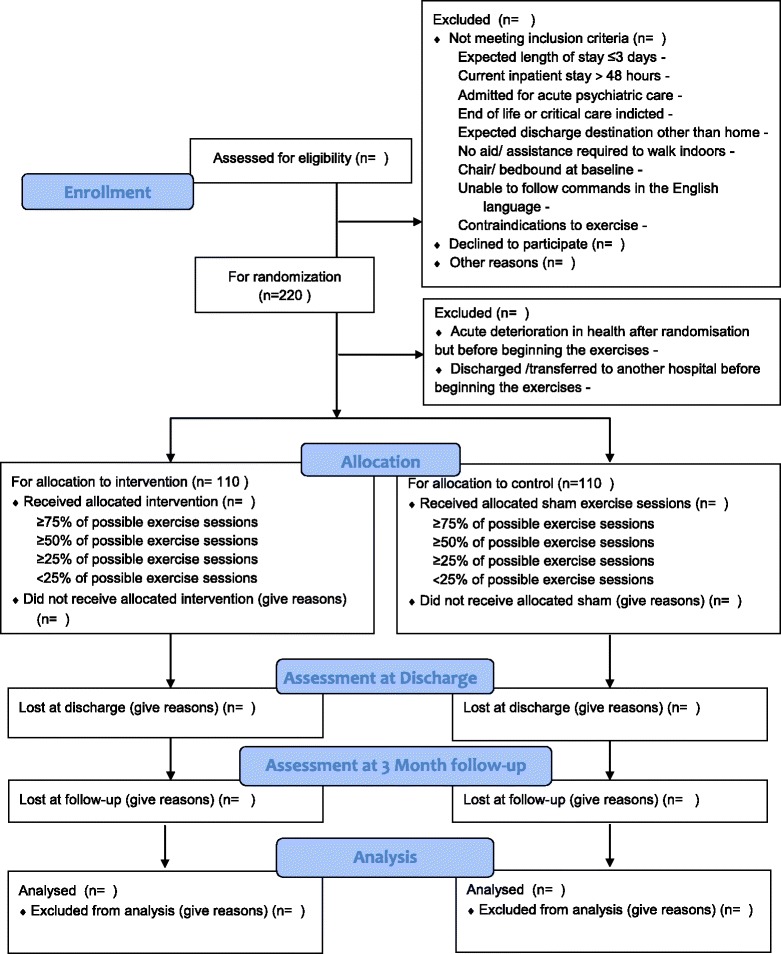
Flowchart with details of the study design and flow of participants

The study is a single blind randomised controlled trial set in an acute 350-bedded teaching hospital. Power calculations based on the results of a pilot study indicated that a sample size of 200 (100 patients in each arm) would be required. To allow for an expected attrition rate of 5 % [18], two hundred and twenty medical patients aged 65 years and over are randomly allocated to either the intervention or sham arm in a ratio of 1:1. (see Fig. 1). The study has been approved by the Clinical Research Ethics Committee of the Cork Teaching Hospitals. (ECM 3 (vv) 13/10/15).

### Selection of participants and allocation

All suitable patients are screened and if eligible, are informed of the study and written consent is sought. The inclusion criteria are: medical patients aged 65 and over, who have been admitted from home and initially planned for discharge home, whose anticipated length of stay is greater than 3 days, and who require a mobility aid or assistance to walk. The exclusion criteria are: patients who have been an in-patient for more than 48 h prior to screening, who are unable to follow commands in the English language, unable to exercise with the assistance of one person only, bed or chair-bound at baseline, admitted with an acute psychiatric condition, require active end-of-life or critical care or when exercise is contraindicated.

To ensure adequate treatment time is given to each patient, recruitment is paused when there are five patients active in the trial. Based on the hospital’s usual length of stay, this usually results in one patient recruited each weekday. If more than one patient is eligible for the study on one day, they are approached in chronological order of admission.

The patients are randomly allocated to either the intervention (APEP) or control group. A computer-generated random allocation sequence is used. Block randomisation is applied (in groups of approximately 50 patients). Post hoc power analysis will be calculated when the first seventy-five patients have completed the trial.

### Roles of the researchers

Randomisation and data entry is completed by the Research Assistant (RA, SO’M). Screening, recruitment, baseline measurements and all exercise sessions are completed by the Principal Investigator (PI, RMcC). The discharge and follow-up assessments are completed by the blinded Research Physiotherapist (RPT, EO’C), who has no involvement in either the allocation or intervention components of the trial.

### Measurements

Patients are assessed within 48 h of admission, at discharge and at three months following discharge. The assessment tools are described in Table 1. Baseline data includes demographics, co-morbidity, medication use and home circumstances.

**Table 1:** Summary of the Measurements used in the Study

Domain	On admission	Discharge and 3 months
Medical Morbidity	Cumulative Illness Rating Scale (CIRS-G [19]);	
	Total number of medications	
Frailty	SHARE- FI [20]	Grip Strength (kgs)
Physical Performance	Short Physical Performance Battery (SPPB) (includes walking speed) [22]	SPPB [22] (includes walking speed)
Falls Efficacy and Self-Reported Functional Ability	Number of Falls and injuries sustained	Number of falls and injuries sustained
	Falls Efficacy Scale – International (FES-I) [25]	Nottingham Extended Activities of Daily Living Scale (N-EADL) [26]
	Nottingham Extended Activities of Daily Living Scale (N-EADL) [26]	
Cognition	6CIT [21]	6CIT [21]
Quality of Life	EQ-5D-5 L [24]	EQ-5D-5 L [24]
Physical Activity	Accelerometers (Stepwatch Activity Monitor, SAM) during hospitalisation only	

The primary outcome measure is length of stay, a key healthcare utilisation metric. The secondary outcome measures includes patient-related measures: changes in physical performance (SPPB, walking speed), and quality of life at 3 months following discharge, differences in physical activity levels between groups in hospital (based on accelerometry data) and re-admission rates over three months. The baseline assessment is designed to capture frailty, co-morbidity and disability. Measurements that are appropriate and quick to administer have been chosen to limit patient fatigue.

### Measurements of Co-morbidity and frailty

The Cumulative Illness Rating Scale Geriatrics (CIRS-G) has been chosen as it measures chronic medical illness burden with good reliability and is a validated tool both as an indicator of health status and as a predictor of 18-month mortality and hospitalisation [19]. The CIRS-G has 14 categories, with a 0 to 4 grading system of impairment in each organ system. The score may theoretically range from 0 to 56, with a higher score reflecting greater impairment in several systems.

To measure frailty, the SHARE-FI [20] tool has been chosen as it is a valid and simple measurement of frailty. Five SHARE variables approximating Fried’s frailty definition are used: fatigue, loss of appetite, grip strength, functional difficulties and physical activity. Scores range between 2.7 to 13.4 and the SHARE-FI calculators (gender-specific) are freely available on the web to interpret the level of frailty [20].

The Six-Item Cognitive Impairment Test (6-CIT) will be used to measure cognition as it is quick to administer, its diagnostic accuracy is as high as the Mini-Mental State Examination when used in the acute hospital setting, and it is not sensitive to an educational level and does require advanced language skills [21].

### Measurements of disability

Physical Performance is measured using the Short Physical Performance Battery (SPPB), which includes walking speed [22]. The SPPB has been chosen as it is quick, practical and safe to use with this population. The scores range from 0 (unable to stand independently,) to 12 (independent tandem balance for 10 s, able to walk 4 m within 4.82 s and sit to stand 5 times in 11 s). Walking speed is known to be a strong indicator of patients’ physical performance and is an independent predictor of survival and institutionalisation [23]. All patients scoring less than 1 on the SPPB will be eliminated from the study to allow us to detect functional decline while in hospital.

### Measurements of well-being, self-efficacy and self-reported functional ability

Quality of Life will be measured using the EuroQol 5-Domain 5-Level Scale (EQ-5D-5 L) [24] as it is well-used and easy to administer. The five domains assessed are mobility, self-care, usual activities, pain/discomfort, anxiety/depression, and a visual analogue scale, ranging from 0 to 100, to measure self-rated health status [24].

At baseline only, fear of falling is measured using the Falls Efficacy Scale-International (FES-I) [25]. This tool consists of 14 activity-related questions. The questions aim to determine how concerned older adults are about falling while performing these activities on a scale of 0 (not concerned at all) to 4 (very concerned).

The Nottingham Extended Activities of Daily Living Scale (N-EADL) [26] is a self-reported tool to measure the patients’ ability to complete 16 community-based activities. On admission, patients are asked to report their functional ability both pre-morbidly (before the onset of the illness) and on admission (the day before they were admitted) and again, at the three month follow-up assessment. Patients can score 0 (unable to complete the activity with/without help) or 1 (able to complete the score with/without help). This has been used extensively in older adult populations, including patients with stroke and fallers [27,28].

### Measurements of physical activity

All patients with good skin condition at the ankle are asked to wear accelerometers (Stepwatch Activity Monitors, SAM) to measure physical activity (step-count) in the hospital. These devices have been validated in frail older in-patients and can be worn in the shower, helping compliance and successful data collection [29]. They are capable of storing up to seven days of data without interruption using a 15 s epoch. They are attached on the first day of recruitment and worn continuously while in hospital or for the first seven days. All staff are informed of their application. The accelerometry data will be analysed to measure

1. Changes in activity between groups.
2. Levels of physical activity compared to a recently completed observation study (which assisted in identifying those who were at risk/not at risk of functional decline).

### Procedure

#### Intervention and routine care schedule

Both groups receive usual multidisciplinary care. The medical team refer patients to physiotherapy if required. It is delivered, an average of three times weekly, by the clinical ward physiotherapist and will be routine in nature. It consists of assessment, discharge planning, exercise, provision of aids and rehabilitation. Both the control and the intervention groups also receive two augmented, twenty minute to half-hour exercise sessions (tailored to the patient’s endurance), five days per week, delivered on a one-to-one basis by the PI.

#### Consent, assessment and exercise procedure

Upon screening, the medical team are contacted to confirm that there is no medical contra-indication to exercise for the patient. Eligible patients are informed of the study verbally and given a copy of the patient information leaflet. They give written informed consent to the study, including access to their medical notes, assessment at baseline, outcome and follow-up, and the twice daily exercise sessions. If the patient is considered to be cognitively impaired by the medical or nursing staff, the patients’ written consent is augmented by verbal assent from their next of kin. Patients with severe confusion, who are unable to follow commands, or are agitated, are not recruited to the study.

If recruited, patients are assessed (Time 1), and through concealed allocation, randomly allocated to the control group or the intervention group at that time. All recruited patients, who consent to wearing the accelerometer and with good skin condition at the ankle, are fitted with the SAM to measure physical activity in hospital.

For those in the intervention (Augmented Prescribed Exercise Programme, APEP) group, their exercise programme is prescribed to address their physical limitations identified through the assessment. The exercises are chosen to improve strength and balance, core stability, sit-to-stand function, balance (in standing and walking), walking and endurance. The intervention group are also actively encouraged to mobilise while in hospital, with assistance when necessary, and provided with walking aids initially, if required. The sham exercise sessions for the control group are not prescribed but consist of standardised stretching and relaxation exercises.

All exercise sessions begin within 24 h of group allocation and continue until the day before discharge. Verbal consent for each session is sought.

To ensure false step-count does not occur, the accelerometer (SAM) is turned upside-down (in this position, it is unable to record steps) when the patient is exercising at the bedside, i.e., not walking, and returned to the upright position before walking or at the end of the session.

Patient compliance, exercise prescription and session duration is recorded. Within one day of planned discharge, all patients are re-assessed and the accelerometer (SAM) is removed from the patient by the RPT (Time 2; see Table 1). Patients who are deemed for long-term care (as they are unable to manage at home) or for end-of-life care are re-assessed on the date that the decision is made and those results are used.

The patients are reassessed at three months post discharge, by the RPT (Time 3, see Table 1). New onset of illness, physical performance, walking speed, quality of life and self-reported functional ability is measured. Hospital and Accident and Emergency utilisation since discharge is recorded.

### Safety, reporting of adverse events and serious adverse events

The main adverse events anticipated in this study are skin rashes from the accelerometer, and falls, cardiac ischaemia or pulmonary embolism during exercise. All adverse events are recorded using an adverse event recording worksheet, and causality to the study intervention is determined, in consultation with the treating physician, by a study physician (KO’C). The Sponsor’s Clinical Research Supporting Officer is notified electronically, within 24 h, of any serious adverse event that occurs during the trial. From a previous local longitudinal study, the Cork Dementia Study, the in-hospital mortality of this cohort is expected to be approximately two per cent [18]. This predicts approximately 5 deaths of trial subjects. However, the type of exercise involved is similar to usual care, patients with a contraindication to exercise will be excluded at source, and patients who are unwell on a particular day will not exercise.

### Statistical analysis

The results will be analysed and presented as recommended by the CONSORT guidelines [30]. The primary outcome measure will be length of stay. This will be described using Kaplan-Meier “survival” curves and the results between groups will be compared using a log-rank test. Univariate and multivariate linear regression analysis will be used to determine differences in physical activity in hospital and physical performance, quality of life at discharge and three months post-discharge, and re-admission rates at three months.

This analysis will help to define whether a simple physiotherapy-led exercise intervention will shorten length of stay, increase physical activity in hospital, limit functional decline and readmission rates and improve quality of life in frail older hospitalised patients.

## Discussion

This study has been designed to measure the effects of an augmented prescribed exercise programme for frail older hospitalised patients. The study design is based upon results of an earlier pilot study and issues reported from previously published studies. Therefore, this protocol differs from previous studies in three key areas: patient selection, intervention and outcome measurements.

Previous studies included some patients who were fully independently mobile but de Morton et al. [31] found that the intervention was most effective for those requiring an aid or assistance to walk. For this reason, we will exclude those who are independently mobile. For pragmatic reasons, we will also exclude those unable to walk at baseline, i.e., bed or chair bound.

There is strong evidence of low physical activity in hospital [32, 33] and Broderick et al. [34] found that many of the barriers could be addressed easily. For these reasons, we will not only deliver supervised exercise sessions, but in addition, will encourage mobility while in hospital and provide walking aids initially, if required. The exercises are designed to improve physical performance, transfer function, walking, balance and strength in order to maintain functional mobility as much as possible.

Our pilot study [13] showed that there was a considerable difference in physiotherapy contact time with the intervention, possibly introducing a Hawthorne effect. This has been addressed by using a sham intervention for the control group. To the authors’ knowledge, this is the first time that a sham intervention has been included in this type of study.

The intervention will be delivered by a senior physiotherapist. The interventions of previous studies have been delivered by a physiotherapist assistant or a physiotherapy student, under the guidance of a qualified physiotherapist [13,16,17]. However, patients’ physical performance fluctuates in the acute setting. If a qualified physiotherapist delivers the programme, it allows the optimal intervention to be delivered on a daily basis. It will also allow a greater opportunity to advise the patient regarding their independent physical activity in hospital and to address barriers to the intervention delivery such as pain relief.

Up to 27 % of the patients were unable to complete the Timed Up and Go Test in previous studies [16,17]. Therefore, the Short Physical Performance Battery will be used, which was found to be feasible and sensitive to change in a previous pilot study [13]. Length of stay will be used as the primary outcome measure as this is available electronically and absolutely complete data. A high attrition rate is predicted for the three-month follow-up attendance as our study group consist of frailer older adults.

A small number of studies have shown that interventions to increase older medical inpatients’ physical activity can be modestly beneficial. Previous authors discuss issues such as patient selection, intervention type and outcome measures. This protocol has been designed to include the frailer patient, to include a tailored and comprehensive intervention, and to measure the effects with the most valid outcome measure.

## Abbreviations

6-CIT: Six-Item Cognitive Impairment Test
APEP: Augmented prescribed exercise programme
CIRS-G: Cumulative Illness Rating Scale – Geriatrics
EQ-5D-5 L: EuroQol 5-Domain 5-Level Scale
FES-I: Falls Efficacy Scale International
N-EADL: Nottingham Extended Activities of Daily Living Scale
PI: Principal Investigator
RA: Research Assistant
RPT: Research Physiotherapist
SAM: Stepwatch Activity Monitor
SHARE-FI: Survey of Health, Aging and Retirement in Europe Frailty Instrument
SPPB: Short Physical Performance Battery
